# Supplementary material for: Transition Metal-Hyperdoped InP Semiconductors as Efficient Solar Absorber Materials
Source: Nanomaterials (Basel). 2020 Feb 7;10(2):283. doi: 10.3390/nano10020283 (PMC7075147; doi:10.3390/nano10020283)
Supplement: Supplementary file 1 [file nanomaterials-10-00283-s001.pdf]

nanomaterials

Supplementary

Transition Metal-Hyperdoped InP Semiconductors as Efficient Solar Absorber Materials

Gregorio García [1,2,*], Pablo Sánchez-Palencia [1,2], Pablo Palacios [1,3] and Perla Wahnón [1,2]

[1] Instituto de Energía Solar, ETSI Telecomunicación, Universidad Politécnica de Madrid, Ciudad Universitaria, s/n, 28040 Madrid, Spain; p.sanchez-palencia@upm.es (P.S.-P.); pablo.palacios@upm.es (P.P.); perla@etsit.upm.es (P.W.)
[2] Departamento de Tecnología Fotónica y Bioingeniería, ETSI Telecomunicación, Universidad Politécnica de Madrid, Ciudad Universitaria, s/n, 28040 Madrid, Spain
[3] Departamento de Física aplicada a las Ingenierías Aeronáutica y Naval, ETSI Aeronáutica y del Espacio, Universidad Politécnica de Madrid, Pz. Cardenal Cisneros, 3, 28040 Madrid, Spain.
* Correspondence: ggmoreno@etsit.upm.es

Received: 14 January 2020; Accepted: 04 February 2020; Published: date

Table S1. Crystal Structure of TM (TM = Ti, V, Cr, Mn), In and P atoms.

Atom	Space Group	Unit cell parameters
In	123	$a = b = 3.25$ Å<br>$c = 4.95$ Å<br>$\alpha = \beta = \gamma = 90^o$
Ti	187	$a = b = 2.95$ Å<br>$c = 4.69$ Å<br>$\alpha = \beta = 90^o$<br>$\gamma = 120^o$
V	221	$a = b = c = 3.03$ Å<br>$\alpha = \beta = \gamma = 90^o$
Cr	221	$a = b = c = 2.91$ Å<br>$\alpha = \beta = \gamma = 90^o$
Mn	217	$a = b = c = 8.92$ Å<br>$\alpha = \beta = \gamma = 90^o$
P	2	$a = 7.86$ Å<br>$b = 5.50$ Å<br>$c = 11.26$ Å<br>α =73.38º<br>β =90.53º<br>γ = 71,62º

Experimental data take from: https://periodictable.com/Properties/A/CrystalStructure.an.wt.html.

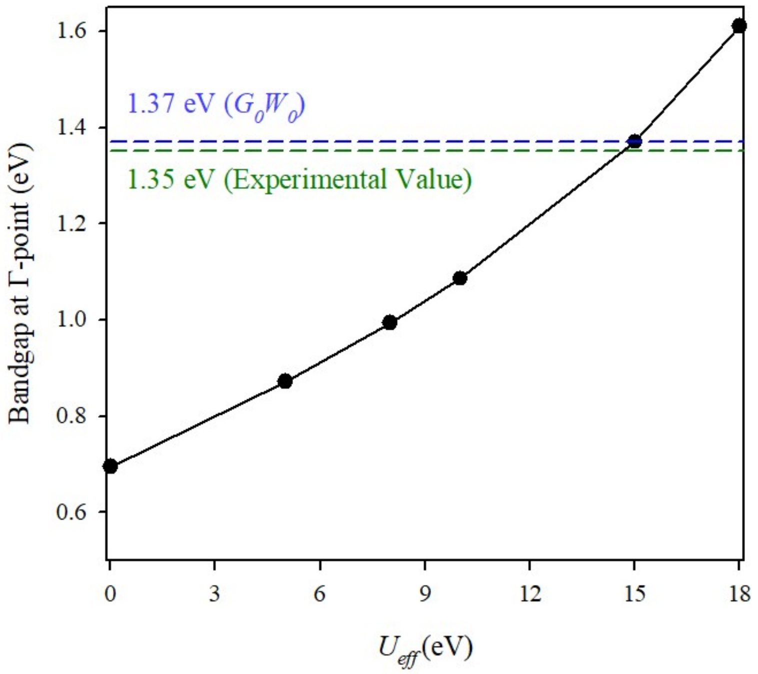

Figure S1. Calculated bandgap at Γ-point for InP by using PBE+*U* formalism (U_{eff} = 5, 8, 10, 15, 18 eV). Crystal structure optimizations were performed using the PBEsol. Blue and green dotted lines stand for G_0W_0 and experimental value.

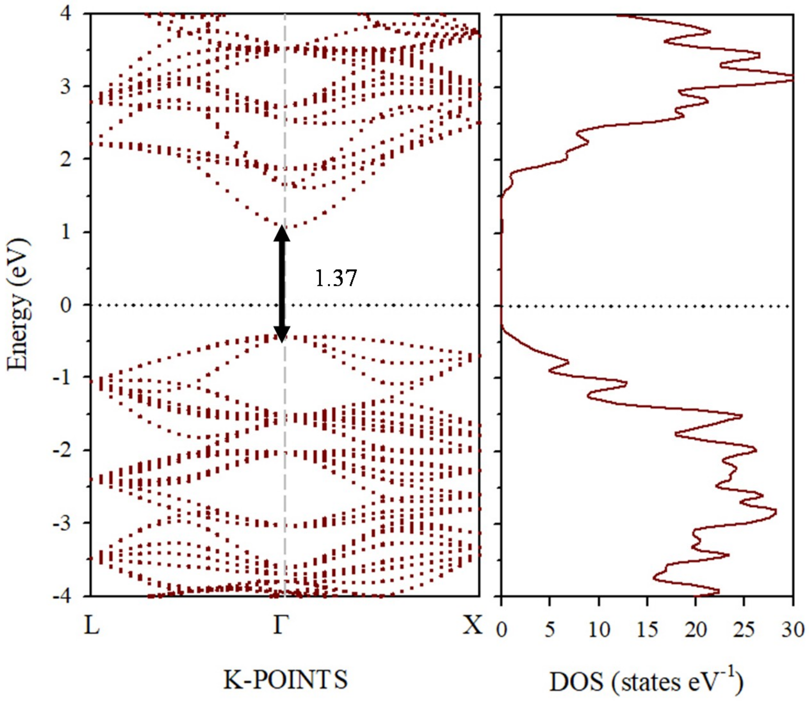

Figure S2. Electronic band structure (left) and Density of States (right) of InP calculated within G_0W_0 approach along to main energy differences at Γ-point. The zero energy has been set to the Fermi level (black dotted line). Spin-up and spin-down bands and DOS are not given separately as both contributions are the same for the native InP material.

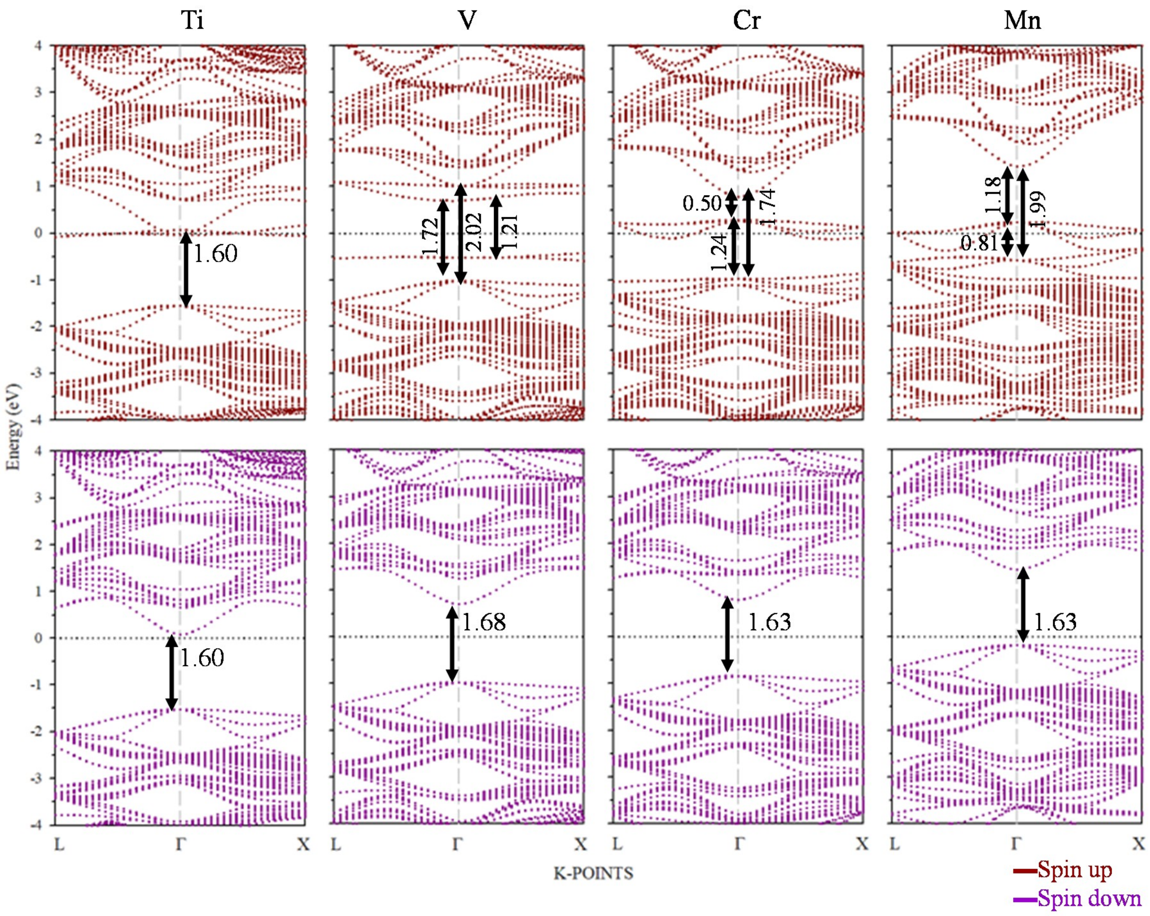

Figure S3. Electronic band structure of TM@InP (TM = Ti, V, Cr, Mn) calculated within G_0W_0 approach along to main energy differences at Γ-point. The zero of energy has been set at the Fermi level (black dotted line).

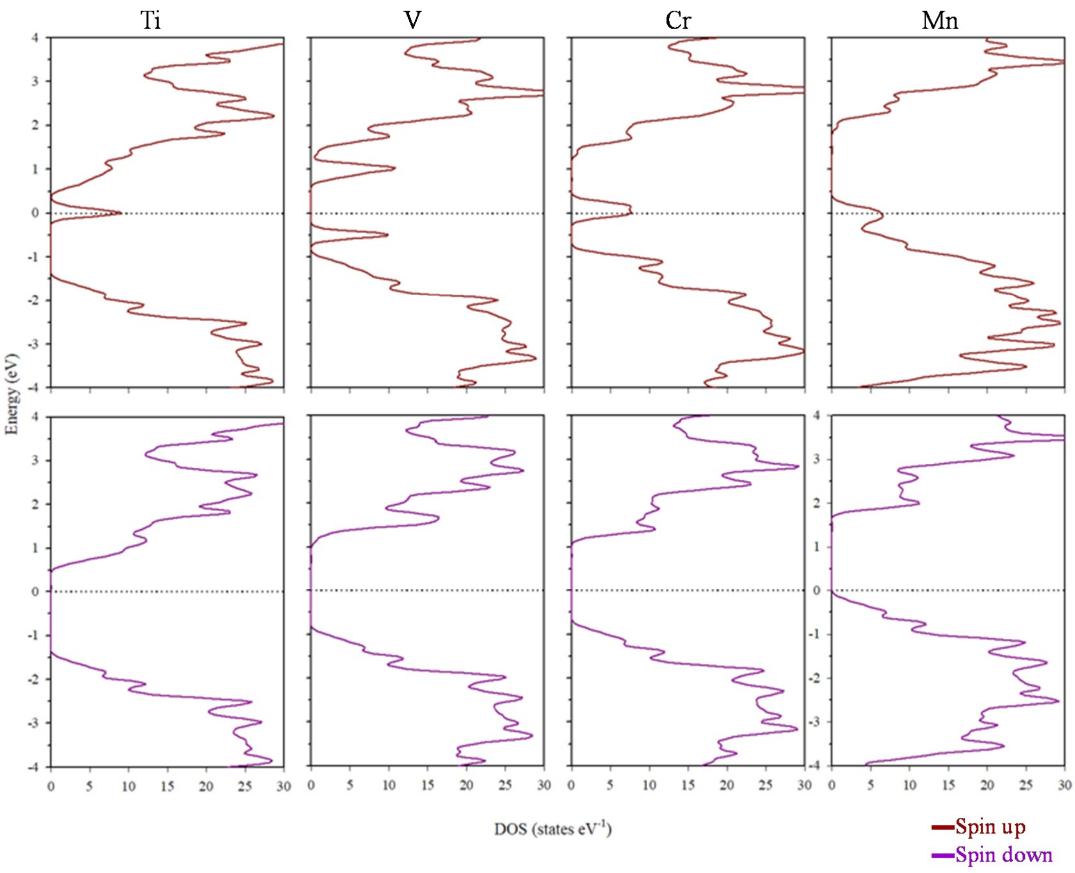

Figure S4. Density of States of TM@InP (TM = Ti, V, Cr, Mn) calculated within G_0W_0 approach. The zero of energy has been set at the Fermi level (black dotted line).
